# Oridonin attenuates atherosclerosis by inhibiting foam macrophage formation and inflammation through FABP4/PPARγ signalling

**DOI:** 10.1111/jcmm.18000

**Published:** 2023-10-31

**Authors:** Ming Zhang, Lianjie Hou, Wanying Tang, Weixing Lei, Huiling Lin, Yu Wang, Haijiao Long, Shuyun Lin, Zhi Chen, Guangliang Wang, Guojun Zhao

**Affiliations:** ^1^ The Sixth Affiliated Hospital of Guangzhou Medical University, Qingyuan People's Hospital Qingyuan China; ^2^ Hengyang Medical School University of South China Hengyang China; ^3^ Guilin Medical University Guilin China; ^4^ Xiangya Hospital, Central South University Changsha China

**Keywords:** atherosclerosis, FABP4, inflammatory response, lipid accumulation, oridonin, PPARγ

## Abstract

Both lipid accumulation and inflammatory response in lesion macrophages fuel the progression of atherosclerosis, leading to high mortality of cardiovascular disease. A therapeutic strategy concurrently targeting these two risk factors is promising, but still scarce. Oridonin, the bioactive medicinal compound, is known to protect against inflammatory response and lipid dysfunction. However, its effect on atherosclerosis and the underlying molecular mechanism remain elusive. Here, we showed that oridonin attenuated atherosclerosis in hyperlipidemic ApoE knockout mice. Meanwhile, we confirmed the protective effect of oridonin on the oxidized low‐density lipoprotein (oxLDL)‐induced foam macrophage formation, resulting from increased cholesterol efflux, as well as reduced inflammatory response. Mechanistically, the network pharmacology prediction and further experiments revealed that oridonin dramatically facilitated the expression of peroxisome proliferator‐activated receptor gamma (PPARγ), thereby regulating liver X receptor‐alpha (LXRα)‐induced ATP‐binding cassette transporter A1 (ABCA1) expression and nuclear factor NF‐kappa‐B (NF‐κB) translocation. Antagonist of PPARγ reversed the cholesterol accumulation and inflammatory response mediated by oridonin. Besides, RNA sequencing analysis revealed that fatty acid binding protein 4 (FABP4) was altered responding to lipid modulation effect of oridonin. Overexpression of FABP4 inhibited PPARγ activation and blunted the benefit effect of oridonin on foam macrophages. Taken together, oridonin might have potential to protect against atherosclerosis by modulating the formation and inflammatory response in foam macrophages through FABP4/PPARγ signalling.

## 1 INTRODUCTION

Atherosclerosis is a disease characterized by lipid accumulation and slowly advancing inflammation in the arterial wall.^1^ This disease is a principal contributor of cardiovascular events, which become the leading cause of mortality globally.^2^ As the major immune cells in vascular lesion, macrophages are crucial for the initiation and development of atherosclerosis. Once trapped in the vessel wall, differentiated macrophages uptake oxidized low‐density lipoprotein (oxLDL) particles, leading to their transformation to foam macrophages. Moreover, the oxLDL particles also induce foam macrophages to produce and secrete a large amount of immune cytokines, resulting in inflammatory response and eventually progression of the plaque.^3^, ^4^ As such, the inflammatory hypothesis and the lipid hypothesis are both considered as the basic risk factors for atherosclerosis.^5^

Over the last decade, most interventions aiming at atherosclerosis control focus on lowing‐lipid.^6^ Statins are widely used recipes in clinic. More than that, several advances are invented to modulate lipid by enhancing cholesterol efflux from macrophage, which is mediated by liver X receptor‐alpha (LXRα) and its downstream elements. Afterwards, cholesterol is removed and transported from macrophage to faeces, thereby improving foam macrophages in lesion.^7^ In spite of this, residual risk is still not satisfactorily solved in atherosclerosis, which remains persistently high incidence rate.^8^, ^9^ Therefore, the establishment of a new therapeutic regime targeting other risk factors of atherosclerosis is greatly important. Currently, accumulating evidence shows an additional role for anti‐inflammation strategy independent of lipid modulation.^9^, ^10^ Inhibition of the key transcription factors, such as Nuclear factor NF‐kappa‐B (NF‐κB), or the expression of downstream cytokines mitigates vascular plaque. Some representative medicine among them have entered clinical trials and even show a reduced risk for atherosclerotic events.^11^ Unfortunately, the side effects of anti‐inflammation strategy, along with the well‐established role of lipid modulation strategy, challenge the application on its own.^12^ In addition, a complex combination of prescription drugs for multifunctional reduce the compliance of patients. Hence, it is necessary to identify a novel and convenient therapy to alleviate lipid and inflammation simultaneously in atherosclerosis.

Oridonin is the bioactive diterpenoid compound from *Rabdosia rubescens*, which is praised as ‘magic grass’ and has a long history of medical consumption in China for many health benefits, including the prevention of cardiovascular disease.^13^, ^14^ Recent studies have shown that oridonin possesses strong therapeutic properties and limited side effects.^15^ For instance, administration of oridonin protects against inflammation, including lipopolysaccharide (LPS)‐induced liver injury, lung inflammation, rheumatoid arthritis and so on.^16^, ^17^, ^18^ There are also studies implying that oridonin can improve the lipid modulation in cancer and non‐alcoholic fatty liver.^19^, ^20^ Moreover, a current clue also points out that oridonin exerts molecular function on stimulating LXRα in hepatocytes.^21^ Motivated by accumulating evidence linking anti‐inflammation and lipid modulation function, we speculate that oridonin may serve as a promising candidate to alleviate both risk factors of atherosclerosis. However, the molecular effect of oridonin against atherosclerosis and the underlying mechanisms remain rarely known, especially in the setting of lipid modulation.

Herein, the present study aimed to observe the effect of oridonin on atherosclerotic plaque and foam macrophage formation. Our results showed that oridonin inhibited atherosclerosis, as it performed double duty on lipid modulation and anti‐inflammation properties in macrophages via fatty acid binding protein 4 (FABP4)/peroxisome proliferator‐activated receptor gamma (PPARγ) pathway.

## 2 MATERIALS AND METHODS

### 2.1 Chemicals and materials

Oridonin (99.89% purity by HPLC), SR9238 and SR‐202 were bought from MCE company. oxLDL and Dil‐oxLDL were purchased from Yiyuan biotechnology. The primary antibody against LXRα was bought from ZEN‐BIOSCIENCE. The primary antibodies against ATP‐binding cassette transporter A1 (ABCA1), ATP‐binding cassette subfamily g member 1 (ABCG1), interleukin‐1β (IL‐1β), nuclear factor NF‐kappa‐B (NF‐κB), beta‐actin and HRP‐conjugated anti‐rabbit IgG secondary antibody were bought from Abcam. The primary antibodies against FABP4 and PPARγ were bought from Proteintech Group. The GAPDH antibody and anti‐rabbit IgG Alexa Fluor® 555‐conjugated secondary antibody were obtained from Cell Signalling Technology. Dimethyl sulfoxide (DMSO) as the vehicle of oridonin was bought from MP Biomedicals. Oil Red O solution was obtained from Sigma‐Aldrich. Foetal bovine serum (FBS), DMEM medium and RPMI 1640 medium were purchased from GIBCO. Goat serum for blocking and DAPI staining solution were obtained from Solarbio.

### 2.2 Animals

This animal experiment was approved by the Ethics Committee at the Sixth Affiliated Hospital of Guangzhou Medical University (animal ethical approval number: LAEC‐2022‐014). The animal experiment was performed according to the Guide for the Care and Use of Laboratory Animals. Six‐week‐old male apolipoprotein E‐deficient (ApoE^−/−^) on a C57BL/6 background (18–22 g) were purchased from Aniphe Biolaboratory Inc. Mice were housed in a 12‐h light/dark environment under the temperature of 25 ± 1°C, with free access to water and feed. Twelve mice were randomly divided into two groups. For atherosclerosis modelling, the mice fed high‐fat diet food with 60% fat Kcal (Medicinence, Jiangsu) for a procedure of 12 weeks. Six mice from oridonin group were intraperitoneally injected with 5 mg/kg of oridonin every day starting from the 5th week. Six mice from control group were intraperitoneally injected with vehicle control (1% DMSO and phosphate buffer solution). At the end of the 12th week, the mice were anaesthetized with isoflurane and sacrificed. The whole aorta and aortic root were collected for the histopathological experiment. The blood was collected for lipid analysis using commercial kit (Solarbio).

### 2.3 Cell culture

Mouse macrophage Raw 264.7 cell line was cultured in DMEM supplemented with 10% FBS. Cells were maintained in a humidified atmosphere to 70%–80% confluence at 37°C with 5% CO_2_. Afterwards, the cells were challenged by 0.1% DMSO (blank control) or oridonin for indicated time.

### 2.4 Cell counting kit 8 assay

A cell counting kit 8 assay (CCK‐8, MCE, USA) was used to determine the cell viability influenced by oridonin. In brief, Raw 264.7 macrophages were seeded in 96‐well plates and allowed to adhere for 24 h. Different concentrations (0, 5, 10, 20 and 40 μM) of oridonin were added to the plates for 24 h. Subsequently, cells were incubated with CCK‐8 solution for another 2 h at 37°C. The absorbance was detected at 450 nm with a microplate reader (SPECTRO Star Nano, BMG Labtech).

### 2.5 Oil Red O staining

Raw 264.7 macrophages were seeded in 12‐well plates and allowed to adhere. OxLDL (50 μg/mL) was added into the cells without or with the stimulation of oridonin (10 μM). After incubation at 37°C with 5% CO_2_ for 24 h, macrophages were washed with phosphate‐buffered saline (PBS) and then fixed with paraformaldehyde for 10 min. Next, Oil Red O solution was used for staining, followed by washing with 50% isopropanol. At last, cells were stained with haematoxylin and photographed. The images were obtained using Olympus BX53 microscope (Tokyo, Japan).

### 2.6 Dil‐oxLDL uptake assay

In order to visualize oxLDL uptake, cells were pre‐treated with oridonin for 30 min and incubated with 40 μg/mL Dil‐oxLDL for 4 h at 37°C protected from light. Then, the cells were fixed with paraformaldehyde for 10 min and stained with DAPI solution for 5 min. Followed by PBS for washing three times, the fluorescence in cells was visualized by an inverted microscope (Zeiss Axio Observer 7, Oberkochen, Germany).

### 2.7 Intracellular cholesterol efflux assay

To detect intracellular cholesterol efflux, a cholesterol efflux assay kit was used (Sigma‐Aldrich). Raw 264.7 macrophages were seeded in 96‐well plate and allowed to adhere for 6 h at 37°C with 5% CO_2_. After washing with FBS‐free RPMI 1640 medium, the labelling mix was added into wells for 1 h at 37°C protected from light according to the instruction. After that labelling mix was aspirated and replaced with the equilibration mix. The plates were then incubated at 37°C overnight protected from light. After the aspiration of equilibration mix, pre‐treated LDL/VLDL‐depleted serum dilution was used as cholesterol acceptors without or with oridonin 10 μM for 8 h. The fluorescence densities of supernatant and cell lysis buffer from samples were measured by Cytation 5 imaging reader (BioTek), based on which the percent of cholesterol efflux was calculated.

### 2.8 Quantitative reverse transcription PCR

After the cells were challenged by oxLDL for 12 h (assays for ABCA1, ABCG1, LXRα and FABP4) or 2 h (assays for inflammatory cytokines), the total RNA was obtained using RNAiso plus reagent in conformance with the instructions (Takara Bio Inc.). RNA concentration and purity were measured by means of a NanoDrop One device (Thermo Fisher Scientific). After that, the total RNA (1000 ng) was reverse‐transcribed into the cDNA using a PrimeScript™ RT reagent kit with gDNA Eraser (Takara Bio Inc.). Next, quantitative reverse transcription PCR (RT‐qPCR) procedure was carried out by SYBR Green Pro Tag qPCR Kit (Accurate Biology). The threshold cycles (CT) were measured by a BioRad CFX96 system. The mRNA expression was determined using the 2^−△△^CT method. Gene GAPDH was used as an internal control. Primers related were synthesized by Tianyi Huiyuan company with sequences listed in Table 1.

**TABLE 1:** Primer sequences for RT‐qPCR.

Genes	Sequences (5′‐3′)
ABCA1	GCTTGTTGGCCTCAGTTAAGG (forward)
	GTAGCTCAGGCGTACAGAGAT (reverse)
ABCG1	CTTTCCTACTCTGTACCCGAGG (forward)
	CGGGGCATTCCATTGATAAGG (reverse)
LXRα	CTCAATGCCTGATGTTTCTCCT (forward)
	TCCAACCCTATCCCTAAAGCAA (reverse)
IL‐6	CCAAGAGGTGAGTGCTTCCC (forward)
	CTGTTGTTCAGACTCTCTCCCT (reverse)
TNF‐α	GACGTGGAACTGGCAGAAGAG (forward)
	TTGGTGGTTTGTGAGTGTGAG (reverse)
IL‐1β	GCAACTGTTCCTGAACTCAACT (forward)
	ATCTTTTGGGGTCCGTCAACT (reverse)
SR‐A1	GCACAATCTGTGATGATCGCT (forward)
	CCCAGCATCTTCTGAATGTGAA (reverse)
LOX‐1	CAAGATGAAGCCTGCGAATGA (forward)
	ACCTGGCGTAATTGTGTCCAC (reverse)
FABP4	AAGGTGAAGAGCATCATAACCCT (forward)
	TCACGCCTTTCATAACACATTCC (reverse)
GAPDH	AGGTCGGTGTGAACGGATTTG (forward)
	TGTAGACCATGTAGTTGAGGTCA (reverse)

### 2.9 Western blotting assay

After the cells were challenged by oxLDL for 24 h, samples were lysed in RIPA buffer containing phenylmethanesulfonyl fluoride (PMSF) on ice (Beyotime). After centrifugation, the total protein was extracted from supernatant. The protein was separated by SDS‐PAGE and transferred to polyvinylidene fluoride (PVDF) membrane (Millipore). The membrane was blocked by skimmed milk for 1 h and then incubated with the primary antibodies overnight at 4°C. The membranes were washed three times and then incubated in HRP‐conjugated anti‐rabbit IgG secondary antibody at room temperature for 1 h. Next, the protein bands were developed using an Ultra High Sensitivity ECL Kit (MCE) and scanned by a BioRad ChemiDoc imaging system. GAPDH or beta‐actin protein was used as an internal control. The densitometry was analysed by ImageJ software (National Institutes of Health).

### 2.10 Immunofluorescence staining

To observe the location of NF‐κB, immunofluorescent staining was used on Raw 264.7 macrophages. Cells were seeded in 12‐well plates at a density of 3 × 10^5^ every well. After 0.1% DMSO or oridonin (10 μM) pre‐treatment for 0.5 h, macrophages were treated with oxLDL (20 μg/mL) for two more hours at 37°C 5% CO_2_. Subsequently, macrophages were fixed with 4% paraformaldehyde for 10 min. 0.1% Triton X‐100 was used for permeabilization. After washed with PBS, macrophages were blocked with 1% goat serum for 30 min. Primary antibody against NF‐κB (ab16502, 1:200) was used to incubate cells at 4°C overnight. AlexaFluor 555 anti‐rabbit secondary antibody (#4413, 1/1000) was used to visualize NF‐κB staining. Nuclei were also co‐stained using DAPI staining. Images were obtained using a Zeiss Axio Observer 7 inverted microscope.

### 2.11 Screening for potential targets of oridonin against atherosclerosis

The information regarding target genes of oridonin was collected using the database CtdBbase (http://www.ctdbase.com/), HIT 2.0 (http://hit2.badd‐cao.net/) and HERB (http://herb.ac.cn/). Next, the genes related to atherosclerosis disease were collected from database CtdBbase (http://ctdbase.org), DisGeNet (www.disgenet.org) and Phenopedia (https://phgkb.cdc.gov/). The common target genes connecting oridonin and atherosclerosis were visualized using Venn diagrams (https://bioinfogp.cnb.csic.es/tools/venny/index.html). The annotation and function of these genes were obtained from Genecards (https://www.genecards.org/).

### 2.12 Protein–protein interaction network construction and cluster analysis

To construct protein–protein interaction (PPI) network for exploring the core gene of oridonin against atherosclerosis, the intersectional genes were imported into STRING database (https://string‐db.org). The interaction threshold was set to ‘0.4’.

### 2.13 Biological function annotation and pathway analysis

To further assess the anti‐atherosclerosis mechanism of target genes in oridonin, an online database DAVID (https://david.ncifcrf.gov/) was used to analyse the intersectional genes for GO (Gene Ontology) functional enrichment analysis. The top ten items of biological process (BP), molecular function (MF) and cellular component (CC) in the GO analysis were presented with a bar chart. In addition, we also performed KEGG (Kyoto Encyclopedia of Genes and Genomes) enrichment analysis for annotating signalling pathways. The significantly enriched pathways were shown in a bubble graph.

### 2.14 RNA sequencing

Total RNA was extracted from oxLDL‐induced foam macrophages treated with or without oridonin using RNAiso plus. Each group had three biological replicates. RNA integrity was performed using the Bioanalyzer 4150 system (Agilent Technologies). The libraries were generated as published previously (MustSeq, an alternative approach for multi‐plexible strand‐specific 3′ end sequencing of mRNA transcriptome, confers high efficiency and practicality). The libraries were sequenced on Illumina Platform Novaseq 6000 S4 sequencer. Quality control of raw data was processed through Trimmomatic v0.39. Files of gene model annotation were based on Ensembl website. Differential expression analysis was processed using the DESeq2 R package. The differentially expressed gene was defined with a *p* < 0.05 and fold change ≥1.5.

### 2.15 Statistical analysis

All experiments were performed independently in triplicate. The data was presented as mean values ± standard deviation. An unpaired, two‐tailed Student's *t*‐test was used to compare data from two groups. And statistical analyses for multiple groups using one‐way anova followed by the Bonferroni post hoc test. Statistical differences were considered significant for a *p* value less than 0.05.

## 3 RESULTS

### 3.1 Oridonin protects against atherosclerosis progression

**FIGURE 1:**
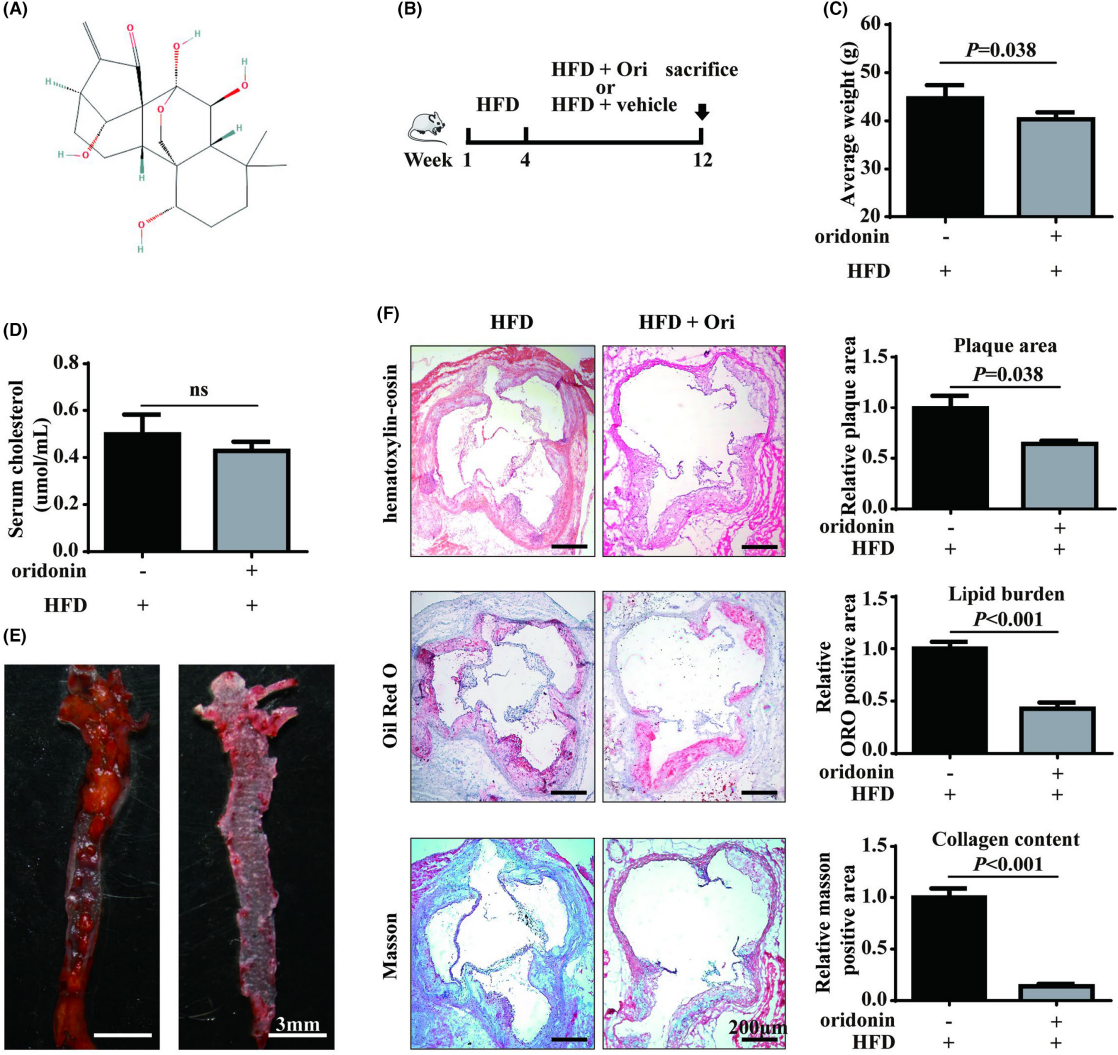
Effects of oridonin on atherosclerosis in ApoE^−/−^ mice. (A) Chemical structure of oridonin (downloaded from PubChem database https://pubchem.ncbi.nlm.nih.gov/). (B) Study design in ApoE^−/−^ mice fed with high‐fat diet food. (C) The average body weight of mice intraperitoneally injected with oridonin or vehicle. (D) The serum level of total cholesterol from mice was determined. (E) The representative staining of the whole aorta with Oil Red O. (F) The cross sections of aortic arch were stained with haematoxylin–eosin staining, Oil Red O staining and masson staining. Quantitation of area at aortic arch was analysed respectively. Data are presented as the means ± SD (*n* = 6 per group). HFD, high‐fat diet food; Ori, oridonin.

To investigate whether oridonin protected against the progression of atherosclerosis, two groups of ApoE^−/−^ mice fed with high‐fat food were injected with oridonin or vehicle (Figure 1A,B). After 12 weeks, the body weight in oridonin‐treated mice was slightly decreased, while the levels of serum total cholesterol were not significantly reduced compared with the control group (Figure 1C,D). The further histopathological experiments showed that the mice in oridonin‐treated group had a relatively smaller plaque along the whole aorta and in the aortic root (Figure 1E,F). These results indicated that oridonin protected against atherosclerosis progression in vivo.

### 3.2 Oridonin ameliorates the foam macrophage formation and inflammatory response

**FIGURE 2:**
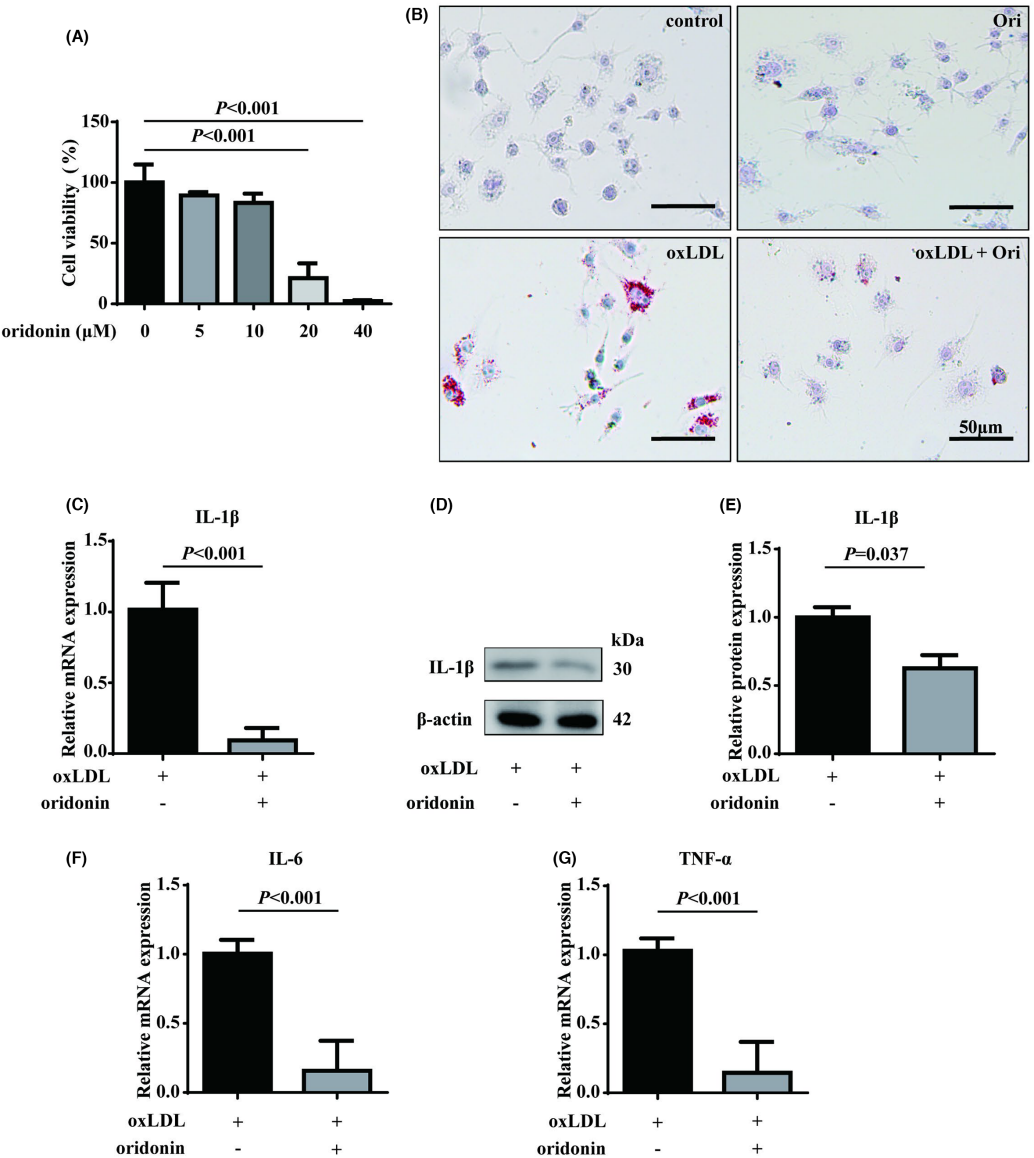
Oridonin alleviates lipid accumulation and inflammatory response in foam macrophages. (A) CCK‐8 assay was employed to evaluate cell viability. (B) Oil Red O staining used to evaluate the lipid accumulation in macrophages treated with oxLDL (50 μg/mL) without or with 10 μM oridonin. The magnification of each panel is 200×. Representative images of three independent assays are shown. (C) The relative mRNA expressions of IL‐1β evaluated by RT‐qPCR in foam macrophages treated with or without oridonin. (D) The protein expressions of IL‐1β was evaluated using western blotting. Representative images of three independent assays are shown. (E) Quantitation of protein expressions of IL‐1β was shown. The relative mRNA expressions of IL‐6 (F) and TNF‐α (G) evaluated by RT‐qPCR in foam macrophages treated with or without oridonin. Data were mean ± SD from at least three independent experiments. RT‐qPCR, quantitative reverse transcription PCR.

To further evaluate the effect of oridonin in vitro, its cytotoxicity in different concentrations was initially evaluated on Raw 264.7 cells. CCK‐8 assay revealed that only high doses of oridonin (over 10 μM) affected the viability of cells (Figure 2A). Therefore, the concentration of 10 μM was used for subsequent experiments. Oil Red O staining was used to analyse the formation of foam macrophages. As shown in Figure 2B, we found that oxLDL treatment resulted in accumulated lipid in macrophages, while oridonin alleviated the lipid staining apparently. Moreover, it is noteworthy that the formation of foam macrophages produces the inflammatory response, thereby promoting the progression of atherosclerosis. The pro‐inflammatory cytokines, including IL‐1β, Interleukin 6 (IL‐6) and tumour necrosis factor‐alpha (TNF‐α), have been regarded as the risk factors in atherosclerosis.^11^, ^22^, ^23^ Thus, we evaluated the expression of these cytokines in foam macrophages and found that IL‐1β, IL‐6 and TNF‐α were significantly reduced by oridonin treatment (Figure 2C–G). Together, we demonstrated that oridonin protected against the lipid accumulation and inflammatory response during the formation of foam macrophages.

### 3.3 Oridonin improves cholesterol efflux in macrophages, but not cholesterol influx

**FIGURE 3:**
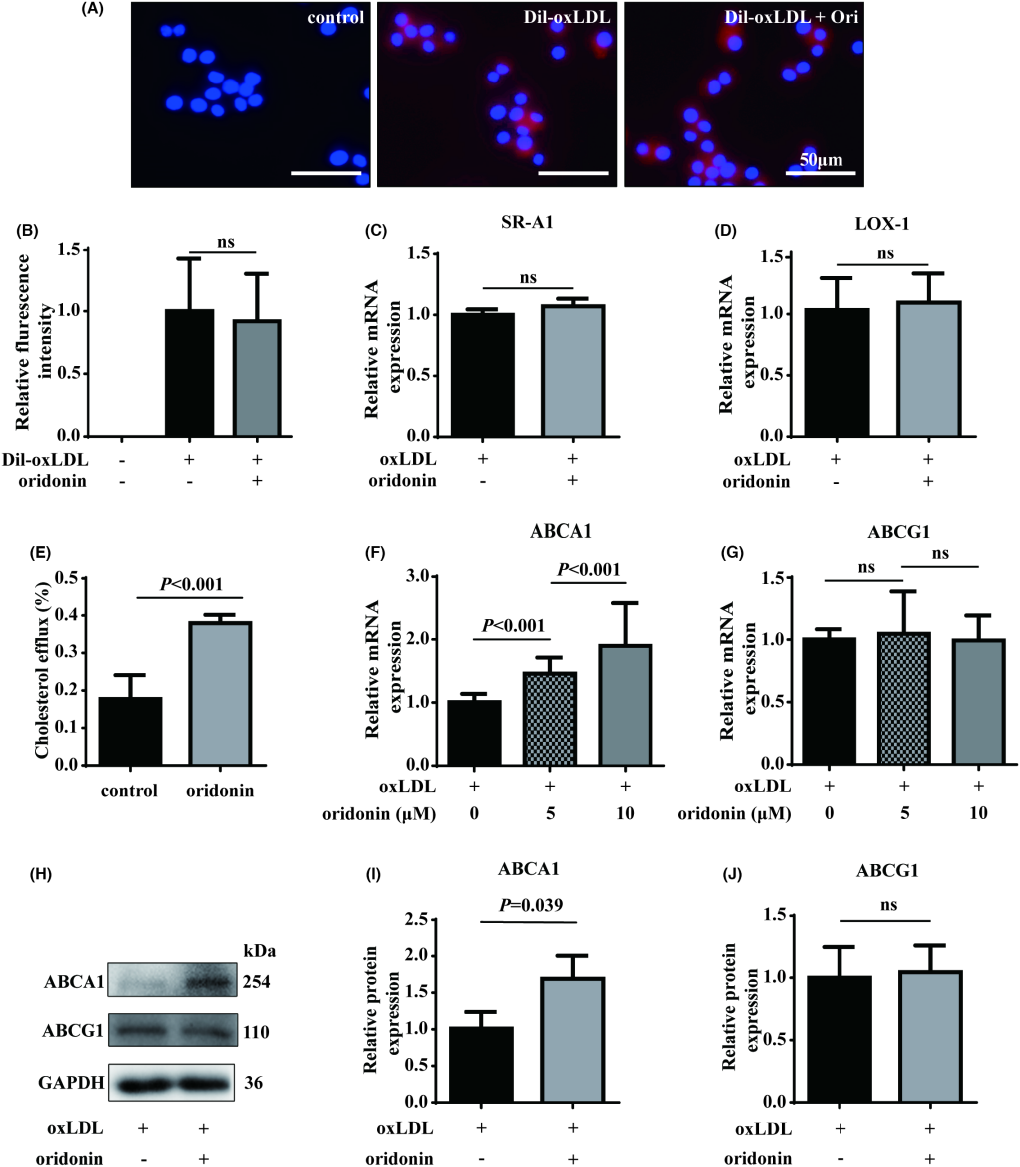
Oridonin increases intracellular cholesterol efflux assay mediated by ABCA1. (A) Fluorescence images of Dil‐oxLDL representing cholesterol influx in macrophages pre‐treated with oridonin. (B) The comparison of fluorescence intensities in Dil‐oxLDL‐treated macrophages. (C, D) The mRNA expression of SR‐A1 and LOX‐1 accessed by RT‐qPCR analysis. (E) Intracellular cholesterol efflux assay as Raw 264.7 macrophages were treated without or with oridonin (10 μM) for 8 h. (F, G) The mRNA expression of ABCA1 and ABCG1 evaluated by RT‐qPCR in macrophages treated with oxLDL and oridonin (0, 5, 10 μM), respectively. (H–J) The protein expressions of ABCA1 and ABCG1 evaluated using western blotting, respectively. Representative images of three independent assays are shown. Data were mean ± SD from at least three independent experiments. ns, no significance; Ori, oridonin; RT‐qPCR, quantitative reverse transcription PCR.

The formation of foam macrophages can result from the increased influx or/and decreased efflux of cholesterol.^24^ To elucidate the mechanism of oridonin‐mediated lipid transportation, we first evaluated the cholesterol influx by Dil‐oxLDL uptake assay. As a result, there was no obvious change in the Dil‐oxLDL amount as the macrophages were pre‐treated with oridonin (Figure 3A,B). In line with that, RT‐qPCR assay did not show significant difference on the expression of receptors for oxLDL uptake including the lectin‐like oxidized LDL receptor 1 (LOX‐1) and the SR‐related and CTD‐associated factor 1 (SR‐A1) (Figure 3C,D). These results demonstrated that oridonin did not alter cholesterol influx in this setting. Next, the cholesterol efflux assay revealed that oridonin significantly enhanced cholesterol efflux in Raw 264.7 macrophages (Figure 3E). ABCA1 and ABCB1 are both main molecular pumps for cholesterol efflux.^25^ Interestingly, oridonin only increased the mRNA and protein expression of ABCA1, but not ABCG1 (Figure 3F–J). Taken together, we found that oridonin ameliorated the foam macrophage formation through ABCA1‐mediated cholesterol efflux.

### 3.4 Network pharmacology analysis identifies LXRα, NF‐κB and PPARγ as functional genes

**FIGURE 4:**
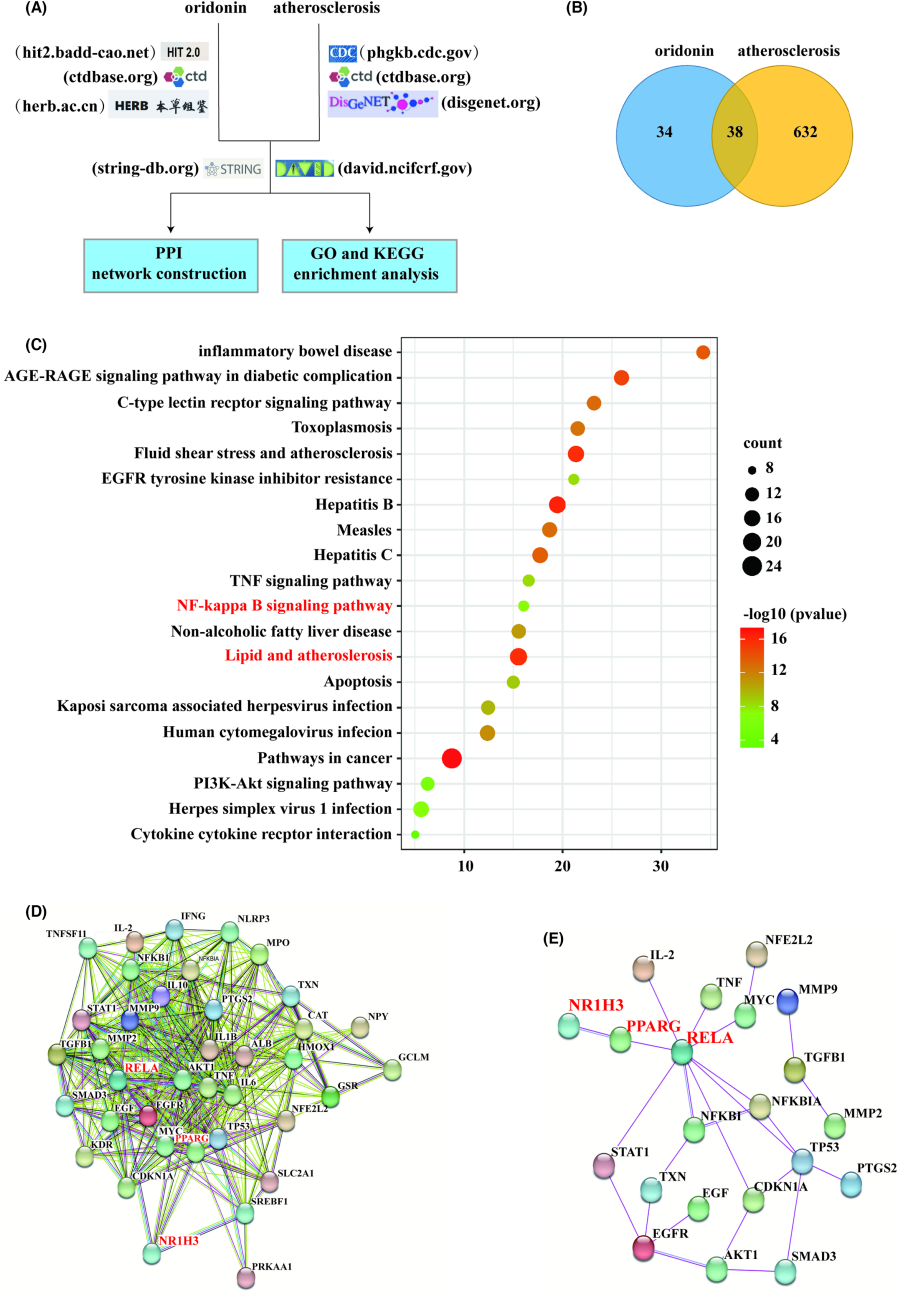
Network pharmacology analysis to predict the mechanism of oridonin against atherosclerosis. (A) Flow chart showing network pharmacology analysis performed in this study. (B) Venn diagram presenting the intersectional target genes of oridonin against atherosclerosis. (C) The bubble diagram of KEGG enrichment analysis showing the top 20 significant pathways. (D) PPI network comprising intersection of identified target genes of oridonin against atherosclerosis. (E) The core PPI network of identified target genes rigidly proved by experiments.

To explore the potential functional genes in oridonin against atherosclerosis, we initially performed a network pharmacology analysis based on current knowledge (Figure 4A). Totally, 670 target genes related to atherosclerosis were obtained by overlapping the information from CtdBbase, DisGeNet and Phenopedia (Figure S1A, Data S1). Then, based on the reviewed pharmacology data, 38 intersectional genes of oridonin against atherosclerosis were retrieved (Figure 4B, Data S2). According to the annotation from Genecards, most of these intersectional target genes were in regard to regulation of inflammation response. Besides, we also noticed that several lipid‐related genes, such as LXRα (encoded by NR1H3) and Sterol Regulatory Element‐Binding Transcription Factor (SREBF1), were included.

To establish elucidate the functions and the enriched pathways of these intersectional genes, GO and KEGG pathway enrichment analyses were performed. As a result, 453 GO terms were significantly involved, including 371 BP terms, 24 CC terms and 58 MF terms. Among them, the top 10 significant terms were visualized in Figure S1B. Furthermore, KEGG pathway analysis indicated that the top 30 statistically significant pathways (Figure 4C). Besides the pathways associated with inflammatory response including the NF‐κB pathway, the ‘lipid and atherosclerosis’ pathway with 18 gene counts was also involved.

To elucidate the regulative genetic network of oridonin against atherosclerosis, a PPI analysis was constructed with the intersectional genes. A network of 38 nodes with 446 edges was obtained (Figure 4D). The average node degree was 23.5. To narrow the profile, we reconstructed a core PPI network based on the results of proved biological experiments. In this network, NF‐κB P65 subunit (encoded by RELA) with the most edges was highlighted, besides another gene as NF‐κB subunit (encoded by NFKB1). Notably, we found that PPARγ, a master regulator of lipid and inflammation, connected NF‐κB with LXRα (Figure 4E). Therefore, we speculated that oridonin might affect atherosclerosis against lipid and inflammation pathways, in which LXRα, NF‐κB and PPARγ might have a vital role.

### 3.5 Oridonin enhances LXRα and inhibits NF‐κB in foam macrophages

**FIGURE 5:**
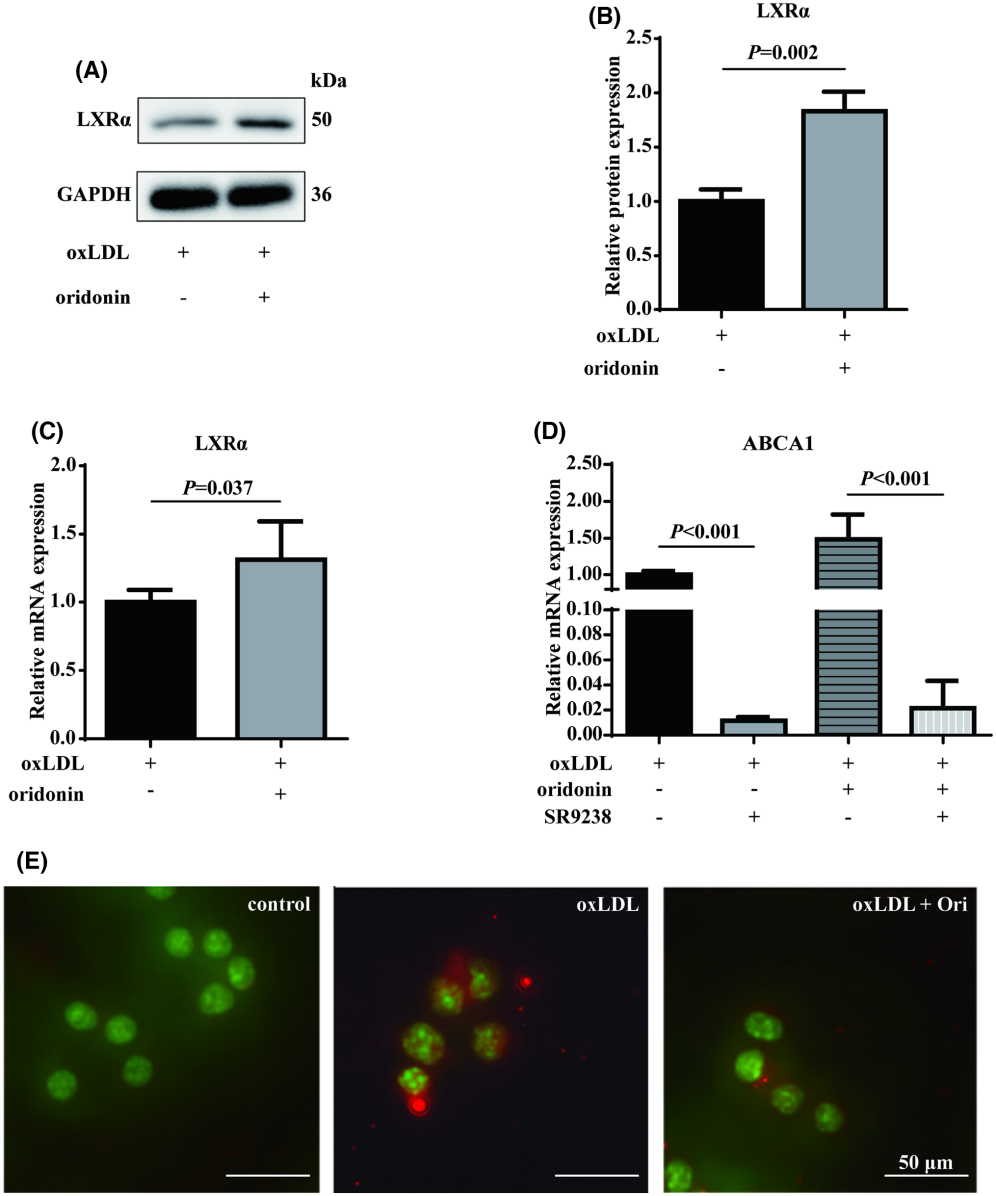
Oridonin increases LXRα expression and inhibits NF‐κB translocation. (A, B) The relative protein expressions of LXRα were evaluated by western blotting. Representative images of three independent assays are shown. (C) The relative mRNA expressions of LXRα evaluated by RT‐qPCR. (D) The relative mRNA of ABCA1 in oxLDL‐treated Raw 264.7 macrophages incubated with SR9238 (10 μM). (E) The NF‐κB translocation to nuclei measured using immunofluorescence staining. The magnification of each panel is 400×. Data were mean ± SD from at least three independent experiments. Ori, oridonin; RT‐qPCR, quantitative reverse transcription PCR.

To verify the main functional genes predicted by network pharmacology, we measured the expression of LXRα in the foam macrophages treated by oridonin. The data showed that mRNA and protein expression of LXRα was increased by the addition of oridonin to the foam macrophages (Figure 5A–C). As we all know, LXRα plays an important role in regulating cholesterol efflux by ABCA1.^26^ We demonstrated that the inverse agonist of LXRα, SR9238, could significantly reversed the expression of ABCA1 induced by oridonin (Figure 5D). Meanwhile, NF‐κB was a ubiquitous transcription factor for multiple pro‐inflammation cytokines. Upon translocation to nucleus, NF‐κB initiates transcription and activates inflammation. Therefore, we used immunofluorescence to locate NF‐κB, demonstrating that pre‐treatment of oridonin inhibited the nuclear translocation of NF‐κB, which was induced by oxLDL (Figure 5E). Thus, these results reinforced the notion that oridonin might regulate the activation of LXRα and NF‐κB to act on the biological function.

### 3.6 Oridonin inhibits foam cell formation and inflammation by activating PPARγ

**FIGURE 6:**
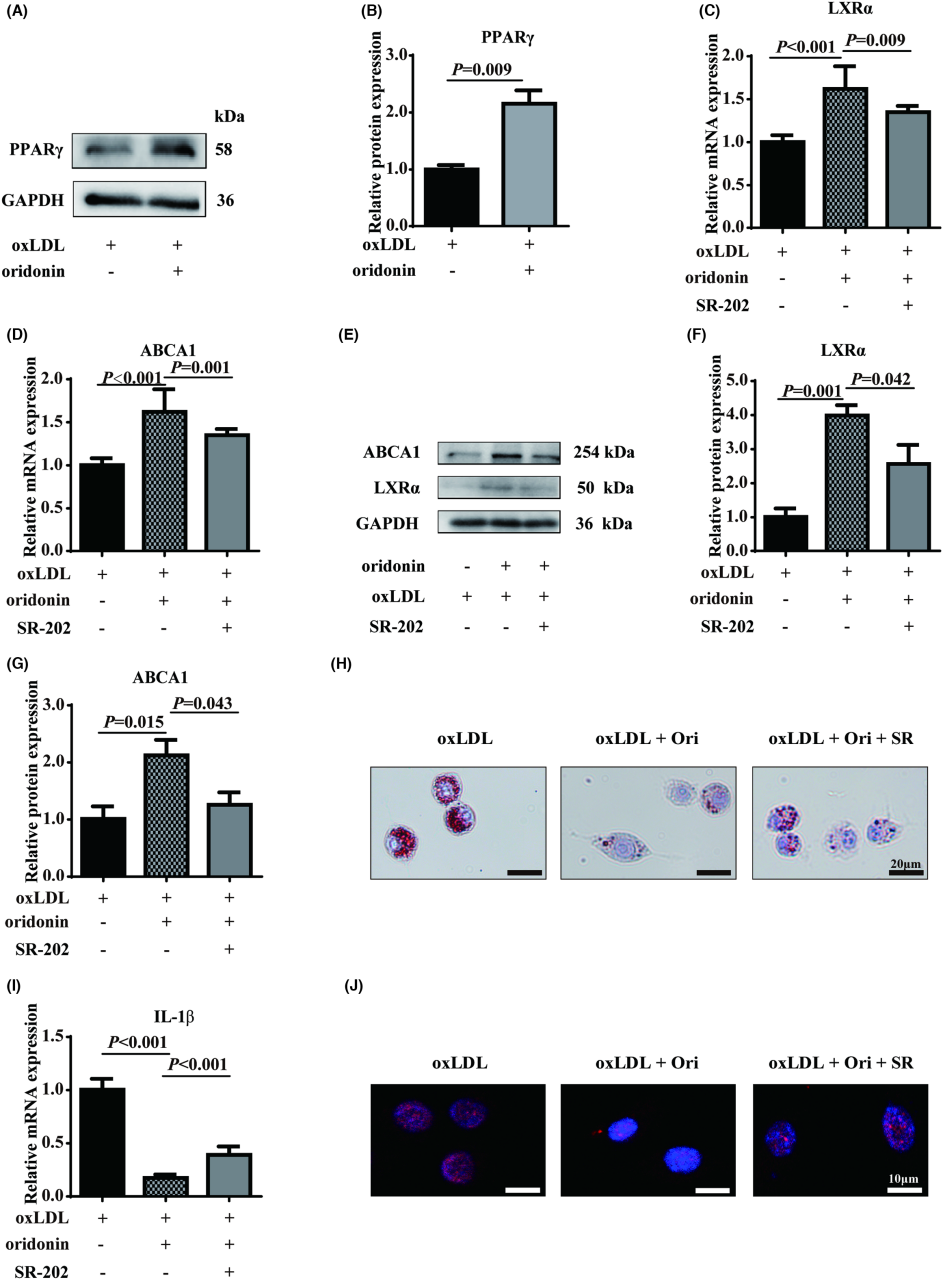
Effects of oridonin on PPARγ activation. (A, B) The protein expression of PPARγ in oxLDL‐treated foam macrophages assessed by western blot. (C, D) The relative mRNA expressions of LXRα and ABCA1 in foam macrophages pre‐incubated with the PPARγ antagonist SR‐202. (E–G) The protein expression of LXRα and ABCA1 determined in foam macrophages pre‐incubated with SR‐202. (H) Oil Red staining in foam macrophages pre‐incubated with SR‐202. (I) The relative mRNA expressions of IL‐1β in foam macrophages pre‐incubated with SR‐202. (J) The immunofluorescence assay for NF‐κB in foam macrophages pre‐incubated with SR‐202 translocation was assessed. The magnification of each panel is 400×. Data were mean ± SD from at least three independent experiments. Ori, oridonin; RT‐qPCR, quantitative reverse transcription PCR; SR, SR‐202.

It is worth noting that PPARγ plays an important role in atherosclerosis and regulates the activity of LXRα and NF‐κB.^27^ Here, we also confirmed the PPARγ was the hub gene regulating LXRα and NF‐κB using network pharmacology analysis. As expected, we found that the expression of PPARγ was significantly increased in foam macrophages treated by oridonin (Figure 6A,B). To further determine the role of PPARγ in foam macrophages treated by oridonin, we used the PPARγ antagonist (SR‐202) to pre‐treat Raw 264.7. As a result, the RT‐qPCR and western blotting analyses demonstrated that PPARγ inhibition significantly reversed the oridonin‐induced expression of LXR‐α and ABCA1 (Figure 6C–G). In addition, PPARγ inhibition could significantly mitigate the protective effect of oridonin on foam macrophage formation. (Figure 6H). Furthermore, PPARγ inhibition also impaired the effect of oridonin on inflammatory response, resulting in increased levels of cytokines and NF‐κB activation (Figure 6I,J, Figure S2A,B). Collectively, the results demonstrated that oridonin protected against the foam macrophage formation and inflammation partly, at least, through PPARγ signalling.

### 3.7 Oridonin attenuates foam cell formation and inflammation via suppressing FABP4

**FIGURE 7:**
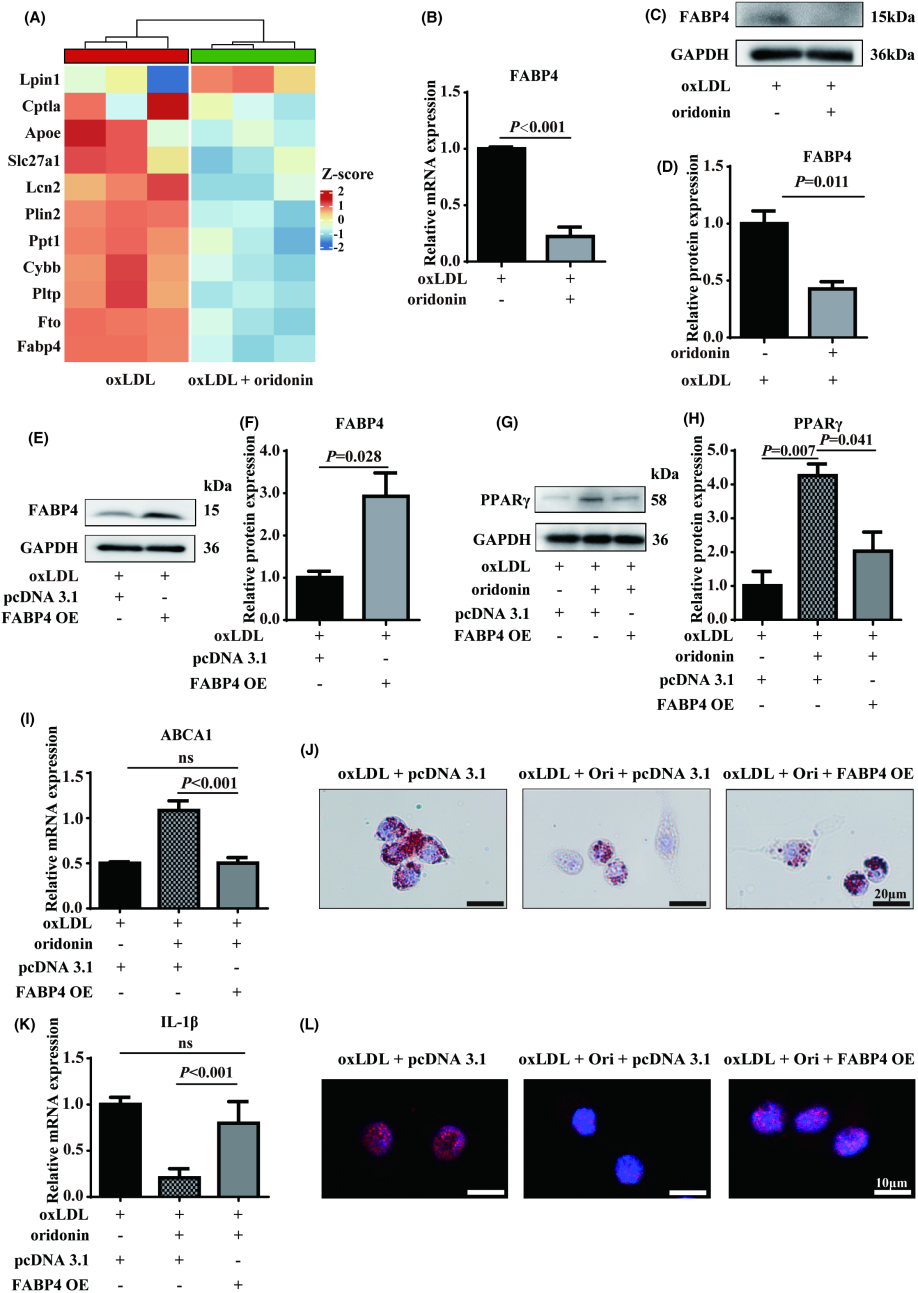
Oridonin attenuates foam cell formation and inflammation via FABP4/PPARγ pathway. (A) Heat map of lipid metabolism genes regulated in response to oridonin. Red shading indicates up‐regulation and blue indicates down regulation. (B) The mRNA expression of FABP4 in foam macrophages treated with or without oridonin assessed by RT‐qPCR. (C, D) Representative western blot analysis of FABP4 expression in foam macrophages treated with or without oridonin. (E, F) The protein expression of FABP4 in foam macrophages transfected with FABP4‐overexpression plasmid. (G, H) The oridonin‐induced protein expression of PPARγ in foam macrophages transfected with or without FABP4‐overexpression plasmid. (I) The oridonin‐induced mRNA expression of ABCA1 in foam macrophages transfected with or without FABP4‐overexpression plasmid. (J) Oil Red staining of foam macrophages treated with oridonin, in combination with FABP4 overexpression. (K) The oridonin‐induced mRNA expression of IL‐1β in foam macrophages transfected with or without FABP4‐overexpression plasmid. (L) The immunofluorescence assay for foam macrophages treated with oridonin, in combination with FABP4 overexpression. Data were mean ± SD from at least three independent experiments. OE, overexpression; Ori, oridonin; RT‐qPCR, quantitative reverse transcription PCR.

To further disclose the mechanism of oridonin on foam macrophages, we performed mRNA sequencing for transcriptome analysis. The analysis showed that several differentially expressed genes related to lipid metabolism were significantly changed by oridonin treatment (Figure 7A). Considering the reported interaction of FABP family with PPARγ, as well as its biological role in coordinating both lipid metabolism and NF‐κB activation,^28^ FABP4 was of particular interest for further study. First, the relative mRNA and protein expressions of FABP4 were validated by RT‐PCR and western blotting, which conformed that FABP4 was the dominantly decreased in foam macrophages treated by oridonin (Figure 7B–D). Then, we used pcDNA3.1 plasmid to overexpressed FABP4 in Raw 264.7 cells and vector plasmid as a control (Figure 7E,F). The effect of FABP4 overexpression was assessed in foam macrophages treated by oridonin. As denoted in Figure 7G,H, the oridonin‐induced protein expression of PPARγ was partly abrogated by FABP4 overexpression. The oridonin‐induced expression of LXRα and ABCA1 was also reversed (Figure 7I, Figure S3A). Subsequently, FABP4 overexpression increased the lipid accumulation alleviated by oridonin (Figure 7J). Furthermore, the expression of inflammatory cytokines in the presence of oridonin was also reversed by FABP4 overexpression, as the NF‐κB activation was promoted (Figure 7K,L). Together, we found that oridonin attenuated foam macrophage formation and inflammatory response by regulating FABP4 in foam macrophages.

## 4 DISCUSSION

Given that the lipid accumulation and chronic inflammatory response in vascular macrophages are the predominant risk factors for atherosclerosis development.^22^ In this study, we found that oridonin prevented atherosclerosis progression and exerted dual‐function on foam macrophages. First, oridonin inhibited the progression of atherosclerotic plaque in the ApoE^−/−^ mice fed with high‐diet food. The in vitro data suggested that oridonin regulated the activity of LXRα and nuclear translocation of NF‐κB, resulting in increased ABCA1‐mediated cholesterol efflux, as well as reduced inflammatory response. In addition, we further demonstrated that oridonin could dramatically facilitate PPARγ expression partly via a FABP4‐dependent manner, thereby regulating foam cell formation and inflammatory response. Taken together, these findings revealed the protective value of oridonin against atherosclerosis through FABP4/PPARγ pathway.

To investigate the causative effect of oridonin, we first applied a bioinformatics analysis based on network pharmacology. Recently, network pharmacology is widely used to discover the informed molecular mechanisms of drugs by elucidating their potential target genes.^29^ Most research in this regard is performed to screen out the active components from herbal formulas. In a few cases, this technology can also be carried out to study a specific monomer.^30^ In our study, after screening for the common genes between oridonin and atherosclerosis, several candidate genes regarding lipid modulation and inflammation were identified. The enrichment analysis suggested that oridonin might contain multiple biological function and act on lipid and inflammation pathways. In addition, a PPI network was also constructed, indicating that PPARγ, LXRα and NF‐κB might serve as the core targets in regulatory network. Herein, our bioinformatics prediction using network pharmacology method identified that oridonin might act on lipid and inflammation pathways with PPARγ, LXRα and NF‐κB as potential hub genes.

LXRα, an important regulator of cholesterol efflux in atherosclerosis, has been shown to be down‐regulated in oxLDL‐treated macrophages, as its downstream membrane proteins ABCA1 and ABCG1 were concomitantly decreased. In this study, we found that oridonin increased cholesterol efflux in oxLDL‐treated macrophages. In line with that mRNA and protein content of LXRα and also its downstream ABCA1 were enhanced. In addition, the inhibition of LXRα dramatically diminished the up‐regulation of ABCA1 mediated by oridonin. Interestingly, our result demonstrated that the expression of ABCG1 had no response to oridonin stimulation, which was not parallel with ABCA1. This phenomenon might be due to the underlying difference between ABCA1 and ABCG1, which was consistent with other groups' finding.^31^, ^32^, ^33^ Thus, the beneficial effect of oridonin on lipid accumulation in macrophages might be contributed to the LXRα‐induced ABCA1 up‐regulation, but not ABCG1.

NF‐κB is one of the main transcription factors for regulating cytokines in inflammatory diseases, including IL‐1β, IL‐6 and TNF‐α.^34^, ^35^ The activation of NF‐ĸB can respond to oxLDL in macrophages, thereby causing excessive inflammatory response in foam cell.^36^ On the contrary, the inhibition of NF‐ĸB signalling alleviates atherosclerosis.^37^ Unfortunately, there is still a lack of specific inhibitors of NF‐ĸB approved for clinical use. According to the network pharmacology prediction, we focused on the effect of oridonin on NF‐ĸB as the main regulator in the inflammation pathway. And for the first time, we demonstrated that oridonin inhibited NF‐κB activation in oxLDL‐treated macrophages, which might explain the inhibition of oridonin on the downstream cytokines.

FABP family is traditionally considered as functional proteins coordinating lipid responses.^38^ Recent studies also demonstrate that FABPs exhibited various characteristics, including regulation of inflammation, in many cell types and tissues. Further understanding provides insights into their actions on the development of immunometabolic diseases.^39^ For instance, FABPs can activate NF‐κB and induce IL‐1β production in mice consuming high‐diet food.^40^ Beside, FABPs are essential in diet‐induced obesity. Among these family members, FABP4 performed similar biological function and mainly expressed in macrophages.^41^ Hence, targeting FABPs might act as a novel therapeutic approach for immunometabolic diseases including atherosclerosis. However, the specific inhibitors of FABPs were still scarce. In the present study, mRNA sequencing and further analysis confirmed that oridonin might serve as a qualified inhibitor of FABP4, which was reported for the first time.

What's more, it has been shown that FABPs suppressed PPARγ expression in macrophages and adipocytes, by which FABPs regulate lipid and inflammation.^28^ In addition, the role of PPARγ signalling for improving atherosclerosis is partly dependent on LXRα induction and NF‐κB inhibition.^27^, ^42^ Thus, we focused on PPARγ signalling as the downstream of FABP4. In this study, oridonin suppressed FABP4 and increased PPARγ expression, while overexpression of FABP4 decreased oridonin‐induced action of PPARγ. Collectively, targeting FABP4/PPARγ might be one of the explanation for the intricacies surrounding the multiple functions of oridonin.

Very recently, Wang et al.^43^ demonstrated that oridonin exerted a therapeutic effect towards atherosclerosis through NACHT, LRR and PYD domain containing protein 3 (NLRP3) and nuclear factor E2‐related factor 2 (Nrf2). Interestingly, growing evidence showed that FABP4/PPARγ exerted a regulatory role on NLRP3 and Nrf2. For instance, Huang et al.^44^ reported that pharmacologic inhibition or genetic deletion of FABP4 down‐regulated NLRP3 in mice and macrophages, resulting in loss of pro‐inflammatory pyroptosis. Another study demonstrated that ablation of FABP4 regulated systemic redox capacity and exhibited reduced expression of NLRP3 in macrophages.^45^ PPARγ activation alleviated osteoarthritis through the Nrf2/NLRP3 pathway.^46^ In addition, Lamas et al.^47^ also found FABP4/PPARγ signalling axis regulated the expression of tissue‐protective genes in a Nrf2‐driven manner in monocytes. To sum up, FABP4/PPARγ axis induced by oridonin could be the upstream regulatory pathway of NLRP3/Nrf2 in atherosclerosis.

Nonetheless, there are still some limitations to the study. For instance, our network pharmacology demonstrated that other candidate genes, including SREBF1,^48^ were targets of oridonin against atherosclerosis. In addition, some potential differently expressed genes detected by mRNA sequencing were also found. However, the role of these genes was not verified in the current study. Meanwhile, experiments using other cell lines or genetic animals can offer more evidence to elucidate the contribution and therapeutic significance of oridonin. More studies about oridonin on atherosclerosis are imperative to decipher its role.

**FIGURE 8:**
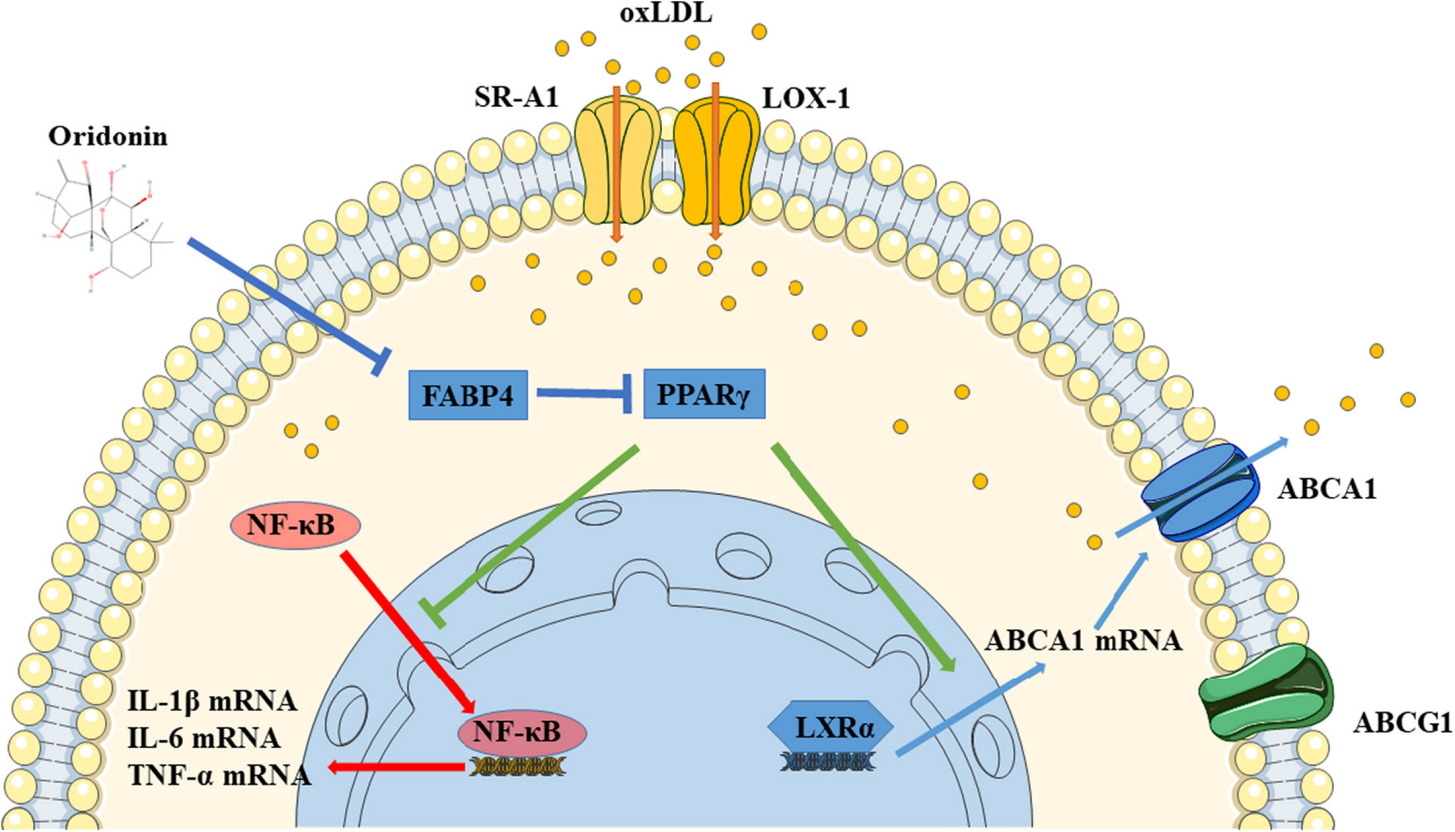
Schematic diagram of the effect of oridonin on foam cell formation and inflammatory response in oxLDL‐treated macrophages.

In conclusion, the present study demonstrated that oridonin attenuated atherosclerosis by suppressing foam macrophage formation and inflammation through FABP4/PPARγ signalling pathway (see Figure 8). Herein, these findings suggest that oridonin may have a novel therapeutic role in the treatment of atherosclerosis.

## AUTHOR CONTRIBUTIONS

**Ming Zhang:** Conceptualization (equal); data curation (equal); project administration (equal); writing – original draft (equal). **Lianjie Hou:** Data curation (equal); investigation (equal). **Wanying Tang:** Methodology (equal); project administration (equal). **Weixing Lei:** Software (equal); validation (equal). **Huiling Lin:** Data curation (equal); methodology (equal). **Yu Wang:** Methodology (equal); project administration (equal). **Haijiao Long:** Formal analysis (equal); investigation (equal). **Shuyun Lin:** Formal analysis (equal); investigation (equal); validation (equal). **Zhi Chen:** Investigation (equal); validation (equal). **Guangliang Wang:** Data curation (equal); writing – review and editing (equal). **Guojun Zhao:** Conceptualization (equal); writing – review and editing (equal).

## FUNDING INFORMATION

This work was funded by the National Natural Science Foundation of China (81870337) and Natural Science Foundation of Guangdong Province (2021A1515010717).

## CONFLICT OF INTEREST STATEMENT

The authors declare that they have no competing interests.

## Supporting information


Figure S1–S3.

Data S1.

Data S2.

## Data Availability

The data that support the findings of this study are available from the corresponding author upon reasonable request.
