# Supplementary material for: Oridonin attenuates atherosclerosis by inhibiting foam macrophage formation and inflammation through FABP4/PPARγ signalling
Source: J Cell Mol Med. 2023 Oct 31;27(24):4155–70. doi: 10.1111/jcmm.18000 (PMC10746953; doi:10.1111/jcmm.18000)
Supplement: Supplementary file 1 — Figure S1–S3. [file JCMM-27-4155-s001.docx]

**SUPPLEMENTAL FIGURES**

**Oridonin attenuates atherosclerosis by inhibiting foam macrophage formation and inflammation through FABP4/PPARγ signaling**

Ming Zhang ^a,1^, Lianjie Hou ^a,1^, Weixing Lei ^b,1^, Wanying Tang ^c^, Huiling Lin ^c^, Yu Wang^a^, Shuyun Lin ^a^, Haijiao Long^a,d^, Zhi Chen ^a^, Guangliang Wang ^a,b^, Guojun Zhao ^a,*^

^a^The Sixth Affiliated Hospital of Guangzhou Medical University, Qingyuan People’s Hospital, Qingyuan, Guangdong, China.

^b^ Guilin Medical University, Guilin, Guangxi, China.

^c^ Hengyang Medical School, University of South China, Hengyang, Hunan, China.

^d^Xiangya Hospital, Central South University, Changsha, China

Corresponding author *

Address for correspondence: zhaoguojun@gzhmu.edu.cn

^1^ These authors contributed equally to the work.

SUPPLEMENTAL FIGURES AND FIGURE LEGENDS

Figure S1


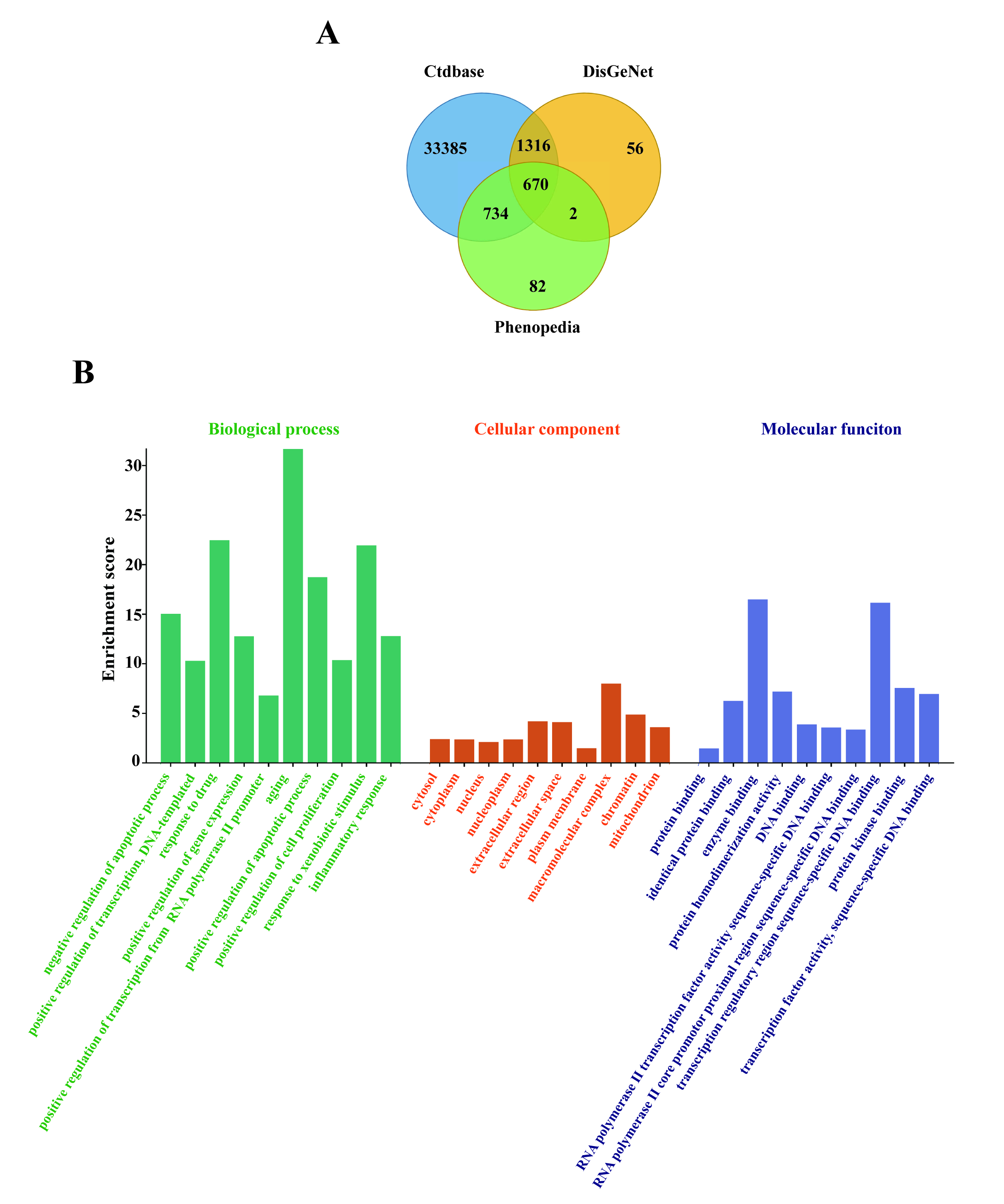


Figure S2


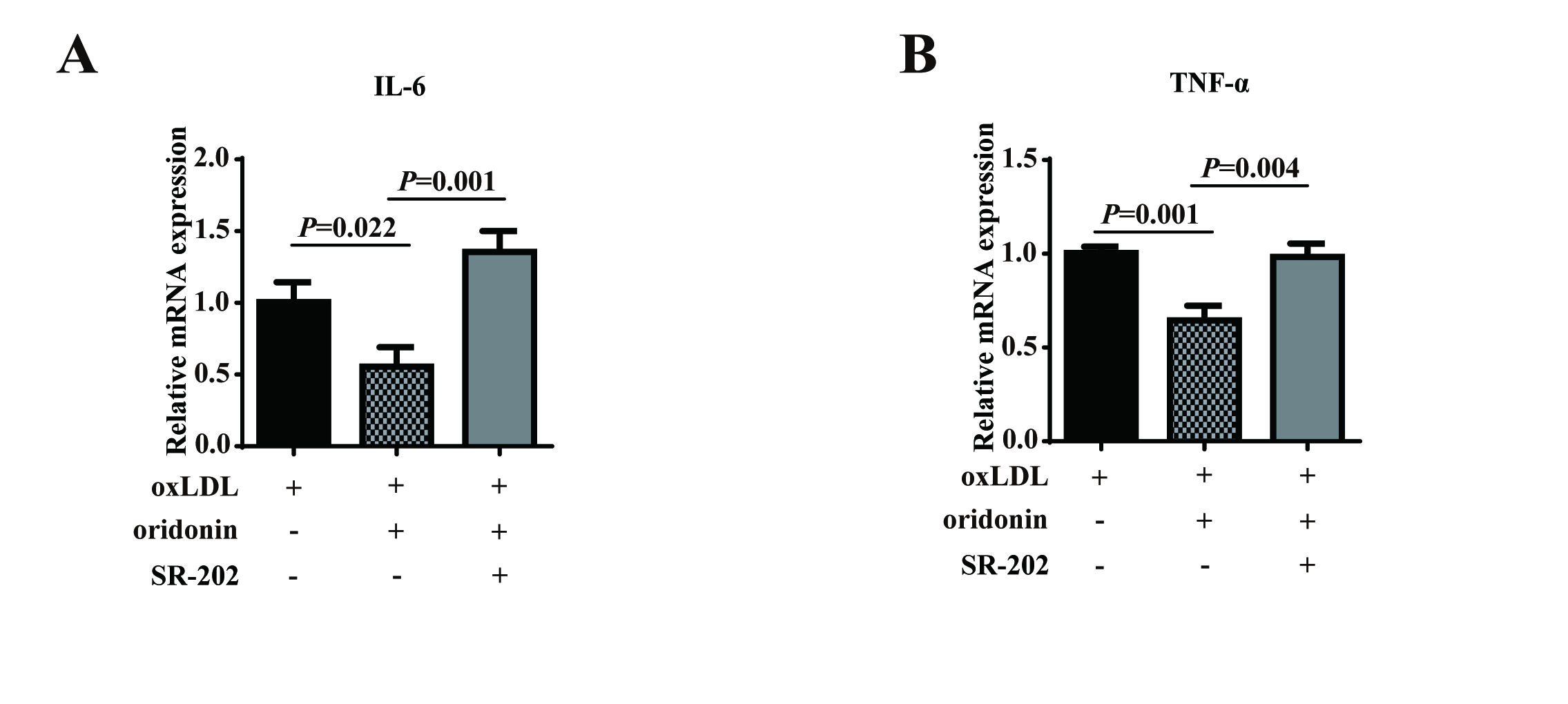


Figure S3


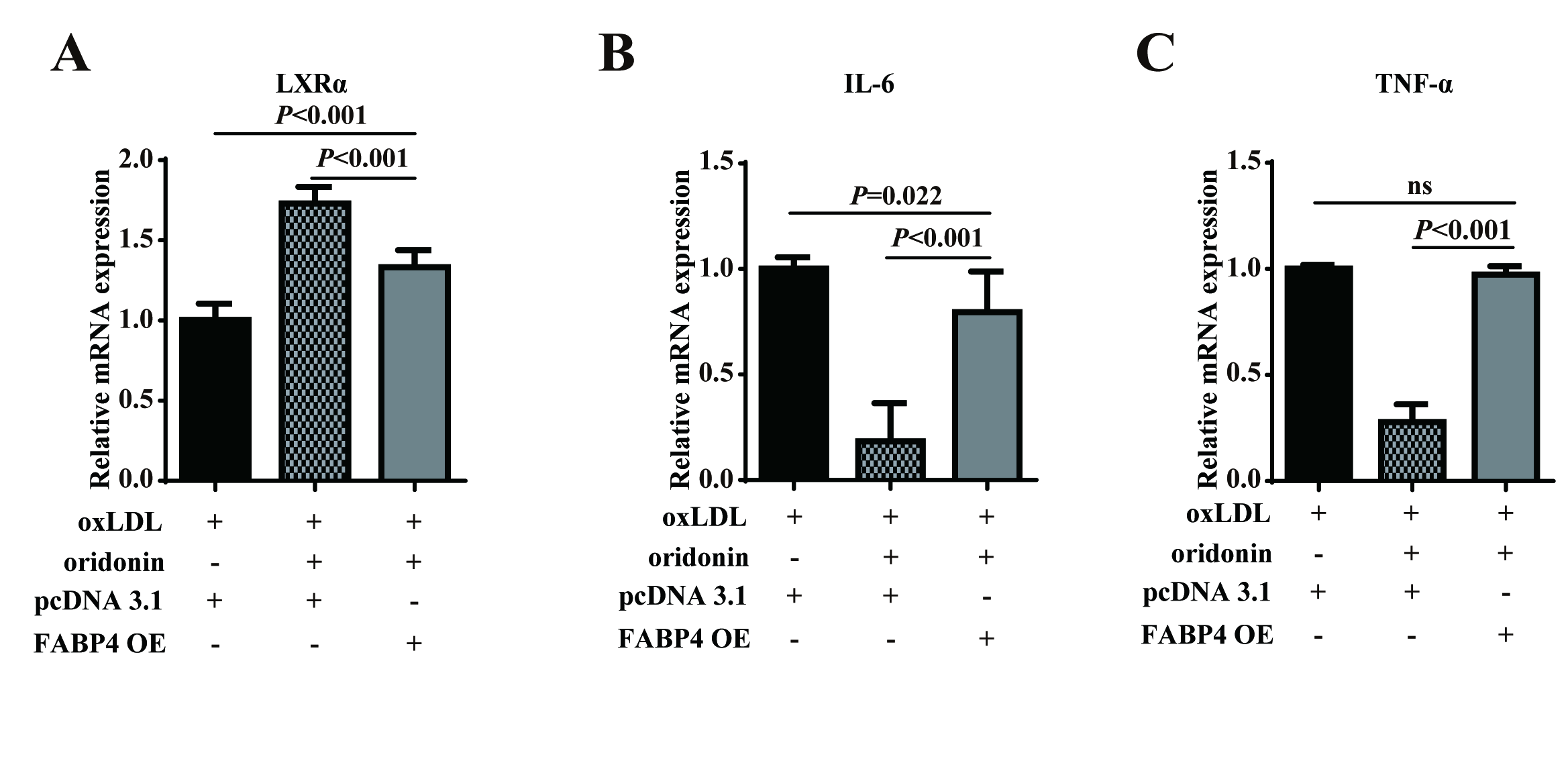


**Figure Legends**

**Figure S1.** Network pharmacology analysis to predict the mechanism of oridonin against atherosclerosis. (A) Venn diagram presenting potential targets genes in the treatment of atherosclerosis. (B) Bar plot diagram of GO enrichment analysis showing the top 10 significant terms, respectively.

**Figure S2.** PPARγ antagonist abrogates the oridonin-induced effect on macrophages. (A-B) RT-qPCR assay in foam macrophages treated with oridonin, in combination with or without PPARγ antagonist, SR-202.

**Figure S3.** Overexpression of FABP4 abrogates the oridonin-induced effect on macrophages. (A-C) RT-qPCR assay in foam macrophages treated with oridonin, in combination with or without overexpression of FABP4. OE, overexpression.
